# Effects of various interventions on non-alcoholic fatty liver disease (NAFLD): A systematic review and network meta-analysis

**DOI:** 10.3389/fphar.2023.1180016

**Published:** 2023-03-29

**Authors:** Xinchen Wang, Xiaoqian Jin, Hancheng Li, Xianyu Zhang, Xi Chen, Kuan Lu, Chenliang Chu

**Affiliations:** ^1^ Department of Pharmaceutical Engineering, College of Food and Pharmaceutical Engineering, Zhaoqing University, Zhaoqing, Guangdong, China; ^2^ Rehabilitation Medicine Department, Zhuhai Hospital Affiliated with Jinan University, Zhuhai, China; ^3^ Department of Epidemiology and Statistics, School of Public Health, Medical College, Zhejiang University, Hangzhou, Zhejiang, China

**Keywords:** non-alcoholic fatty liver disease, non-alcoholic fatty liver, treatment interventions, systematic review, network meta-analysis

## Abstract

**Background:** With the increasing prevalence of obesity and metabolic syndrome, the incidence of non-alcoholic fatty liver disease (NAFLD) is also increasing. In the next decade, NAFLD may become the main cause of liver transplantation. Therefore, the choice of treatment plan is particularly important. The purpose of this study was to compare several interventions in the treatment of NAFLD to provide some reference for clinicians in selecting treatment methods.

**Methods:** We searched Public Medicine (PubMed), Medline, Excerpta Medica Database (Embase), and Cochrane Library from January 2013 to January 2023 to identify randomized controlled trials (RCTs) published in English. The network meta-analysis was performed according to the Preferred Reporting Items for Systematic Reviews and Meta-Analyses (PRISMA) guidelines. Forty-three studies accounting for a total of 2,969 patients were included, and alanine aminotransferase (ALT), total cholesterol (TC), and low-density lipoprotein cholesterol (LDL) were selected as outcome measures for analysis and comparison.

**Results:** We evaluated the results of drug, diet, and lifestyle interventions between the intervention and control groups. Curcumin (CUN) and probiotics (PTC) were selected for medication, the Mediterranean diet (MDED) was selected for special diet (SPD), and various kinds of exercise and lifestyle advice were selected for lifestyle interventions (LFT). The SUCRA was used to rank interventions according to the effect on ALT indicators (SUCRA: PTC 80.3%, SPD 65.2%, LFT 61.4%, PLB 32.8%, CUN 10.2%), TC indicators (SUCRA: PTC 89.4%, SPD 64%, CUN 34%, LFT 36.6%, PLB 17%), and LDL indicators (SUCRA: PTC 84.2%, CUN 69.5%, LFT 51.7%, PLB 30.1%, SPD 14.5%). The pairwise meta-analysis results showed that MDED was significantly better than NT in improving ALT [SMD 1.99, 95% CI (0.38, 3.60)]. In terms of improving TC and LDL, ATS was significantly better than NT [SMD 0.19, 95% CI (0.03, 0.36)] [SMD 0.18, 95% CI (0.01, 0.35)].

**Conclusion:** Our study showed that PTC is most likely to be the most effective treatment for improving NAFLD indicators. Professional advice on diet or exercise was more effective in treating NAFLD than no intervention.

## 1 Introduction

Non-alcoholic fatty liver disease (NAFLD) has become increasingly common. It is defined as bullous fatty degeneration in ≥5% of liver cells that is not related to alcohol or drug use (Li et al., 2021). The incidence rate is high in patients with central obesity, type 2 diabetes, dyslipidemia and metabolic syndrome, and it is also the main cause of chronic liver disease worldwide (Su et al., 2021; Chen et al., 2022; Wing-Sum Cheu et al., 2023). The progression of NAFLD is mainly from non-alcoholic fatty liver disease (NAFL) to non-alcoholic fatty hepatitis (NASH), fibrosis, and cirrhosis, but the public’s understanding of the disease is still very limited (Blot et al., 2023). Despite the obesity epidemic, public health education has not focused on the complications of cirrhosis and even the development of liver cancer.

The treatment principles of NAFLD include identifying high-risk groups, improving metabolic risk factors, detecting the progression of advanced liver disease, and preventing liver injury caused by alcohol, obesity and other factors (Yamada et al., 2022). One study have found that (Sakane et al., 2021) the autophagy ability in white adipose tissue of mice fed with high-fat diet (HFD) was enhanced, and the liver pathology of NAFLD can be improved through autophagy inhibition. Metabolic changes caused by impaired lipid transfer have been speculated to be involved in the molecular pathogenesis of NAFLD (He et al., 2021), and steatosis is a necessary condition for the development of non-alcoholic steatosis. The oxidation of fatty acids in the damaged liver leads to the imbalance of lipid accumulation and redox, and promotes the development of fatty liver disease and insulin resistance. Sun N et al. (Sun et al., 2021)found that the direct KLF16-PPAR *α* pathway was closely related to liver lipid homeostasis and redox balance, and its dysfunction promotes insulin resistance and liver steatosis.

In addition, a higher body mass index is a risk factor for steatosis, and excessive intake of high-calorie foods and obesity contribute to the development of NAFLD (Gao et al., 2017). Therefore, weight loss through diet and exercise is the mainstay of treatment for NAFLD. Studies have shown that regular exercise, such as aerobic exercise, resistance exercise and flexibility training, can improve NAFLD, reduce risk factors inducing NAFLD, such as diabetes, hypertension and cardiovascular disease, and improve liver function and markers of intrahepatic fat in NAFLD (Novelle et al., 2021). The substrates of leptin and adiponectin can regulate energy balance and glucose metabolism through melanocortin activity (Liu et al., 2022). A mouse model (Smn2B/mouse) of surviving motor neuron (SMN) depletion developed severe liver steatosis within less than 2 weeks after birth. The rapid onset of Smn2B/mouse fatty liver provides an opportunity to identify molecular markers of NAFLD (Deguise et al., 2021).

However, for some patients who are unable to achieve weight loss through diet or exercise, drug intervention may be considered. There are many conflicting findings on the efficacy and safety of NASH drug therapy, and the recommendations of management guidelines vary. Therefore, the individual characteristics of patients should be considered to select appropriate drug therapies (Yang et al., 2021; Meijnikman et al., 2022).

The role of the intestinal microbiota in metabolic diseases has received increasing attention (Wang et al., 2021). The imbalance of the microorganisms in the intestine is considered a contributing factor of NAFLD (Qiu et al., 2022). The changes in peripheral and intrahepatic immune responses caused by ecological imbalance accelerate the development of NASH. Ecological imbalance destroys the permeability of the intestinal epithelium, and bacterial antigens enter the portal vein circulation and trigger a downstream inflammatory cascade reaction involving Toll-like receptor 4 (TLR-4) and coreceptors in the liver. In addition, it may also change the intestinal short-chain fatty acids (SCFA), resulting in fatty degeneration and NAFLD (Ramos et al., 2021).

Although research reports on intervention measures to treat NAFLD have emerged continuously, no article has compared the effects of various intervention measures to summarize the adaptive population and characteristics of each measure. Therefore, we selected several intervention methods to treat NAFLD for network meta-analysis (NMA). In terms of medicines, we searched for studies involving curcumin (CUN) and probiotics (PTC). For diet, we chose the Mediterranean diet (MDED), and various exercise or exercise recommendations were searched for. Through the comparison of clinical indicators related to NAFLD, we aim to provide some suggestions for clinicians in the process of choosing intervention measures.

## 2 Materials and methods

### 2.1 Protocol and registration

The systematic review was conducted strictly in accordance with the PRISMA Extension Statement for Reporting of Systematic Reviews Incorporating Network Meta-analyses (Hutton et al., 2015), and the study protocol was registered with the PROSPERO database of systematic reviews (CRD42023401640).

### 2.2 Search strategy and data extraction

We searched Public Medicine (PubMed), Medline, Excerpta Medica Database (Embase), and Cochrane Library from January 2013 to January 2023 to identify randomized controlled trials (RCTs) published in English. We conducted the literature search using the following MeSH terms: “Non-alcoholic Fatty Liver Disease” or “NAFLD” or “Non-alcoholic Steatohepatitis” or “Liver, Non-alcoholic Fatty” and “Curcumin Phytosome” or “Curcumin” or “Phytosome, Curcumin” or “Curcumin Phytosome” or “Probiotics” or “Diet” or “Mediterranean Diet” or “Diets, Mediterranean” or “Mediterranean Diets” or “Lifestyle” or “Exercise” or “Physical Activity” or “Exercise Training” or “motion” or “movement” or “Guideline, Health Planning” or “recommendation” or “Health Planning Recommendation”.

We reviewed the list of references for all eligible articles, study reports and conference reports and searched unpublished literature on the WHO’s International Clinical Research Trials Registry (ICTRP) to obtain additional studies to avoid omissions. After we used EndNote 20 to exclude duplicates, two researchers (XC and XQ) independently screened the titles and abstracts to identify all potentially relevant studies. Two researchers assessed the articles to determine whether they met the inclusion criteria for the study. Differences were resolved through discussion or negotiation with a third party (CL), and experts were consulted if necessary.

### 2.3 Study selection

We determined the eligibility criteria based on PICOS (population, interventions, comparisons outcomes, study designs). The studies included in this review met the following criteria: 1) all study designs were RCTS, 2) participants were diagnosed with NAFLD based on clinical and laboratory data and liver biopsy, which are routine evaluations of the Hepatology Service (de Oliveira et al., 2019), 3) intervention measures included CUN or PTC, the Mediterranean diet, or various types of exercise or exercise recommendations, 4) the control group received no other drug intervention, special diet, exercise, care, or advice, 5) the research results included one of the following indicators: alanine aminotransferase (ALT), total cholesterol (TC), or low-density lipoprotein cholesterol (LDL), and 6) the study language was English. The exclusion criteria were as follows: 1) non-RCTs, 2) review articles, 3) the control group was given special treatment, 4) outcome measures were not correlated with NAFLD, and 5) incomplete data.

### 2.4 Outcome measures

Different from traditional meta-analysis, our NMA does not extract and analyze the relevant results of each study separately but rather extracts, combines and analyzes the results across RCTs. Outcome indicators included ALT to measure liver injury, TC to verify lipid metabolism function, and LDL to detect lipid cholesterol transport.

### 2.5 Quality assessment

The Cochrane Collaboration (Higgins et al., 2011) guidelines were used to evaluate the bias of RCTs included in the analysis, which was mainly evaluated in the following aspects: generation of random sequences, blinding of the outcome data from the implementation, blinding of the subjects and clinical investigators, completeness of outcome data, checking for whether outcomes were present or had been selectively reported, other sources of bias, and whether or not they existed.

### 2.6 Statistical analysis

Continuous variables of NMA were extracted using Stata 20.0 software, and standardized mean differences (SDs) with 95% confidence intervals (CIs) or odds ratios (ORs) with 95% CIs were generated. The statistical heterogeneity criteria for the application of the fixed-effects model were I^2^ < 50% and *p* > 0.01. If these criteria were not met, the random-effects model was used. Heterogeneity among the included studies was quantified by the I^2^ statistic and assessed by Cochran’s Q statistic. I^2^ = 0% indicated no heterogeneity, and I^2^ = 100% indicated maximum heterogeneity. Publication bias and small sample effects were evaluated using funnel plots. Each intervention was ranked using a cumulative ranking curve (SUCRA). Higher SUCRA values indicated a higher likelihood of achieving better therapeutic effects. A matrix was developed to compare all interventions and detect significant SUCRA differences between each pair of interventions. The consistency or inconsistency of these relationships was assessed to enhance the stability of the results. Subgroup analysis of treatment time was performed using Review Manager 5.3 software.

## 3 Results

### 3.1 Literature search and included studies

**FIGURE 1:**
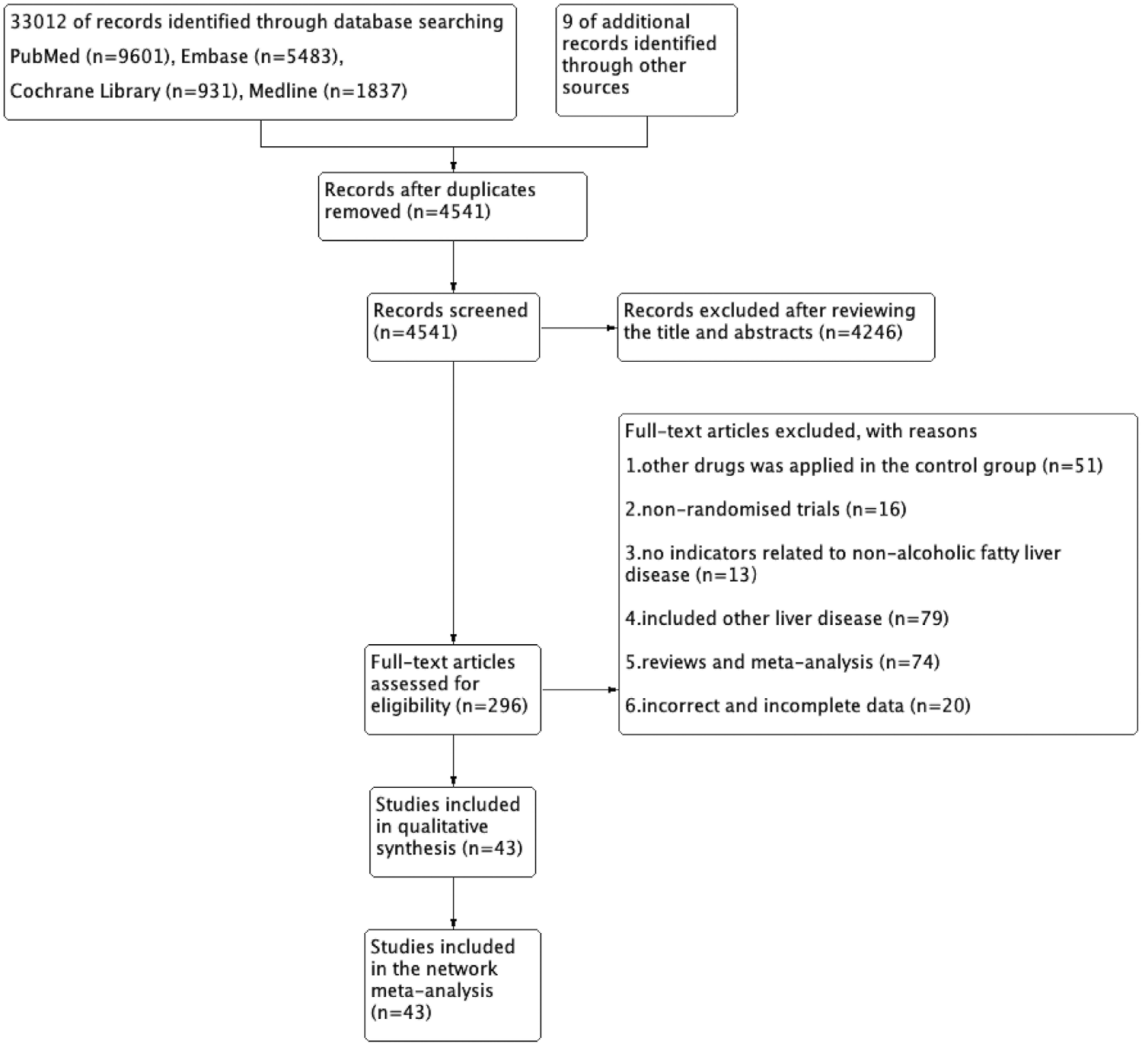
Recruitment flow chart and study design.

A total of 33,012 studies were identified in the initial literature search, and 296 studies were selected for a detailed review after the titles and abstracts were screened. Among them, 253 studies were excluded, including 51 studies in which the control group received other drugs, 16 studies which were non-RCTs, 13 studies in which no indicators were related to NAFLD, 79 studies in which the control group had other liver diseases, 74 studies which were reviews, and 20 studies which had incomplete data. In the end, 43 studies (Pugh et al., 2013; Pugh et al., 2014; Abenavoli et al., 2015; Hallsworth et al., 2015; Dong et al., 2016; Panahi et al., 2016; Rahmani et al., 2016; Zhang et al., 2016; Abenavoli et al., 2017; Cheng et al., 2017; Houghton et al., 2017; Manzhalii et al., 2017; Misciagna et al., 2017; Navekar et al., 2017; Panahi et al., 2017; Dinu et al., 2018; Katsagoni et al., 2018; Wong et al., 2018; Ahn et al., 2019; Cai et al., 2019; Chashmniam et al., 2019; Ghaffari et al., 2019; Ghetti et al., 2019; Mirhafez et al., 2019; Saadati et al., 2019; Campanella et al., 2020; Cicero et al., 2020; Hariri et al., 2020; Nourian et al., 2020; Saberi-Karimian et al., 2020; Çevik Saldiran et al., 2020; Chong et al., 2021; Mirhafez et al., 2021; Mohamad Nor et al., 2021; Alami et al., 2022; Asghari et al., 2022; Chiurazzi et al., 2022; George et al., 2022; Khodami et al., 2022; Li et al., 2022; Mobasheri et al., 2022; Yurtdaş et al., 2022; Ezpeleta et al., 2023) were included in the NMA. The selection process is shown in Figure 1. A total of 2,969 patients with NAFLD were included in our NMA, and the characteristics of the subjects are shown in Table 1.

**TABLE 1:** Definitions of terms in included studies.

Study, year	Patients (no. I/C)	Age years, range or mean ± SD, I/C	Male (No.I/C)	Type of intervention	Intervention	Control		Treatment duration (month) (M)
Ghaffari et al. (2019)	23/23	41.0 ± 8.61/40.3 ± 9.26	21/21	Medication	CUN	Placebo		3 M
Campanella et al. (2020)	57/61	59.07 ± 10.83/59.15 ± 12.76	37/38	Special Diet	MDED	General nutritional counseling and were advised to maintain their lifestyle		6 M
Cicero et al. (2020)	40/40	54 ± 3/53 ± 5	18/19	Medication	CUN	Placebo		2 M
Khodami et al. (2022)	22/21	41.27 ± 11.11/45.81 ± 11.03	15/17	Special Diet	MDED	Usual diet		3 M
Katsagoni et al. (2018)	21/21	41.99 ± 5.56/41.99 ± 4.59	17/13	Special Diet	MDED	Usual diet		3 M
Pugh et al. (2013)	7/6	37.99 ± 2.04/38 ± 4.85	7/3	Lifestyle	ETN	Conventional care		4 M
Pugh et al. (2014)	34/20	33 ± 1.79/33 ± 2.04	22/8	Lifestyle	ETN	Not have any type of treatment		3 M
Houghton et al. (2017)	12/12	54 ± 12/51 ± 16	N/A	Lifestyle	ETN	Not have any type of treatment		3 M
George et al. (2022)	19/23	52.6 ± 11.7/52.1 ± 13.6	8/9	Special Diet	MDED	Low-fat diet		3 M
Ghetti et al. (2019)	20/20	48.3 ± 2.3/50.6 ± 2.3	12/9	Special Diet	MDED plus nutritional orientation	Only nutritional orientation		3 M
Dong et al. (2016)	141/139	56.68 ± 5.33/57.94 ± 5.71	N/A	Lifestyle	ATS	Received only counseling in annual checkups without regular intervention		24 M
Alami et al. (2022)	36/36	47.39 ± 10.29/45.11 ± 9.28	16/16	Special Diet	MDED	Low-sugar diet		6
Yurtdaş et al. (2022)	22/22	13.0 ± 1.99/13.9 ± 2.34	13/13	Special Diet	MDED	Low-fat diet		3
Misciagna et al. (2017)	50/48	30–89	16/10	Special Diet	MDED	Not have any type of treatment		6
Cai et al. (2019)	90/79	35.50 ± 4.42/34.54 ± 6.96	35/23	Lifestyle	ADF	Without any restrictions		3 M
Zhang et al. (2016)	73/74	54.4 ± 7.4/54.0 ± 6.8	22/28	Lifestyle	ETN	Not have any type of treatment		3 M
Hallsworth et al. (2015)	11/12	54 ± 10/52 ± 10	N/A	Lifestyle	ETN	Standard care		3 M
Li et al. (2022)	96/98	42.03 ± 8.53/42.88 ± 8.77	84/82	Lifestyle	ETN	Routine health guidance and follow-up intervention		12 M
Abenavoli et al. (2015)	10/10	46 ± 4.85/28 ± 4.08	6/7	Special Diet	MDED	Not have any type of treatment		6 M
Abenavoli et al. (2017)	20/10	41.99 ± 5.1/24.99 ± 3.83	12/6	Special Diet	MDED	Not have any type of treatment		6 M
Manzhalii et al. (2017)	38/37	44.3 ± 1.5/43.5 ± 1.3	11/16	Medication	PTC	Placebo		3 M
Ezpeleta et al. (2023)	20/20	44 ± 16/44 ± 12	4/4	Lifestyle	ADF	Without any restrictions		3 M
Chiurazzi et al. (2022)	36/32	56.1 ± 9.9/59.8 ± 10.9	16/16	Special Diet	MDED	Not have any type of treatment		3 M
Saberi-Karimian et al. (2020)	27/28	18–70	N/A	Medication	CUN	Placebo		2 M
Hariri et al. (2020)	23/22	40.95 ± 12.24/40.06 ± 13.69	14/12	Medication	CUN	Placebo		2 M
Mohamad Nor et al. (2021)	17/22	54.70 ± 10.19/52.47 ± 16.73	11/17	Medication	PTC	Placebo		6 M
Nourian et al. (2020)	36/33	49.45 ± 1.46/48.23 ± 1.63	N/A	Lifestyle	ATS	Standard care		2 M
Dinu et al. (2018)	20/20	56.4 ± 7.7/53.6 ± 12.2	6/6	Special Diet	MDED	Not have any type of treatment		3 M
Mobasheri et al. (2022)	42/45	35.43 ± 7.881/36.44 ± 8.414	15/15	Lifestyle	Received 8 training sessions based on theory of planned behavior	Received nutritional and physical activity recommendations from their internal specialist and nutritionist		3 M
Navekar et al. (2017)	21/21	42.09 ± 7.23/40.38 ± 9.26	10/8	Medication	CUN	PLB		2 M
Chong et al. (2021)	19/16	57 ± 8/58 ± 7	15/13	Medication	PTC	PLB		2.5 M
Chashmniam et al. (2019)	25/20	46.56 ± 2.25/37.75 ± 3.22	13/14	Medication	CUN	PLB		2 M
Saadati et al. (2019)	27/23	11.5 ± 46.19/10.9 ± 45.13	13/14	Medication	CUN	PLB		3 M
Ahn et al. (2019)	30/35	41.7 ± 12.49/44.71 ± 13.31	15/18	Medication	PTC	PLB		3 M
Rahmani et al. (2016)	37/40	46.37 ± 11.57/48.95 ± 9.78	19/19	Medication	CUN	PLB		2 M
Mirhafez et al. (2019)	33/32	44.8 ± 11.14/40.7 ± 11.83	18/19	Medication	CUN	PLB		2 M
Mirhafez et al. (2021)	35/37	45.0 ± 11.1/43.1 ± 11.6	12/22	Medication	CUN	PLB		2 M
Asghari et al. (2022)	30/30	40.08 ± 7.08/39.27 ± 5.51	20/19	Special Diet	MDED	Healthy eating and weight control advice		3 M
Cheng et al. (2017)	29/29	60 ± 3.5/60 ± 3.4	7/7	Lifestyle	ETN	No-intervention		8.6 M
Çevik Saldiran et al. (2020)	15/16	45.07 ± 9.11/43.75 ± 8.62	6/6	Lifestyle	ETN	Without whole-body vibration		2 M
Wong et al. (2018)	39/39	50.8 ± 8.8/48.1 ± 8.9	23/28	Lifestyle	ATS	Routine care		12 M
Panahi et al. (2016)	44/43	44.98 ± 12.59/47.21 ± 10.29	24/27	Medication	CUN	PLB		2 M
Panahi et al. (2017)	44/43	44.98 ± 12.59/47.21 ± 10.29	24/27	Medication	CUN	PLB		2 M

CUN, Curcumin; PTC, Probiotics; MDED, Mediterranean diet; ETN, Exercise training; ATS, Accept motion theoretical suggestions; ADF, Alternate-day fasting; NT, Not have any type of treatment measure; PLB, Placebo; SD, Standard Deviatio; I/C, Intervention group/Control group.

### 3.2 Results of risk of bias

**FIGURE 2:**
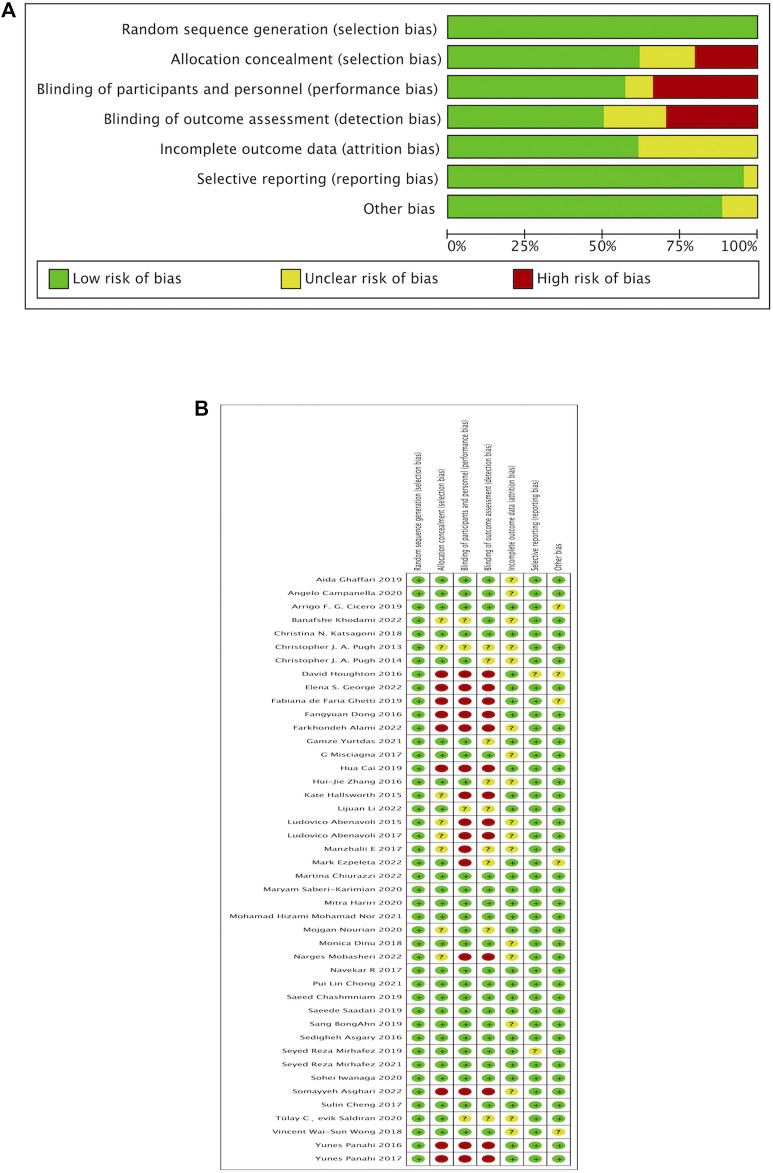
Quality assessment (Cochrane risk of bias tool) for included RCTs. RCT, randomized control study, **(A)** Risk of bias graph, **(B)** Risk of bias summary.

The 43 studies included in the NMA were assessed for risk of bias using methods recommended by the Cochrane Collaboration, and they had varying degrees of bias (In Figure 2).

### 3.3 NMA

We indirectly compared the effects of drugs, diet and lifestyle interventions on the NAFLD-related indicators ALT, TC and LDL. The results showed that there were no significant differences in the NAFLD-related indicators among the various interventions.

We used SUCRA to rank interventions according to the effect on ALT indicators, as shown in Table 2(a). The SUCRA of PTC was 80.3%, which represented the best intervention. The next best intervention was the SPD, with a SUCRA of 65.2%. LFT interventions were ranked third, with a SUCRA of 61.4%. PLB and CUN ranked fourth and fifth, respectively, with SUCRA values of 32.8% and 10.2%. The effect on TC indicators is shown in Table 2(b). The SUCRA of PTC was 89.4%, which represented the best intervention. The next highest was the SPD, with a SUCRA of 64%. CUN ranked third, with a SUCRA of 34%; the SUCRA of LFT and PLB were 36.6% and 17%, respectively. The effect on LDL indicators is shown in Table 2(c). The SUCRA of PTC was 84.2%, which represented the best intervention. The next best intervention was CUN, with a SUCRA of 69.5%. LFT ranked third, with a SUCRA of 51.7%, and PLB and SPD ranked fourth and fifth, respectively, with SUCRA values of 30.1% and 14.5%.

**TABLE 2:** Matrix of pairwise comparison among intervention methods for NAFLD-related indicators.

(a). Matrix of pairwise comparison among intervention methods on ADL (shown as mean difference and 95% confidence intervals)					
	PTC	SPD	LFT	PLB	CUN
SUCRA(%)	80.3	65.2	61.4	32.8	10.2
PTC	0	0.64 (−2.00,3.27)	0.76 (−1.78,3.30)	1.32 (−0.90,3.55)	2.04 (−0.61,4.70)
SPD	−0.64 (−3.27,2.00)	0	0.12 (−1.75,2.00)	0.68 (−0.73,2.10)	1.40 (−0.62,3.43)
LFT	−0.76 (−3.30,1.78)	−0.12 (−2.00,1.75)	0	0.56 (−0.67,1.79)	1.28 (−0.61,3.18)
PLB	−1.32 (−3.55,0.90)	−0.68 (−2.10,0.73)	−0.56 (−1.79,0.67)	0	0.72 (−0.73,2.17)
CUN	−2.04 (−4.70,0.61)	−1.40 (−3.43,0.62)	−1.28 (−3.18,0.61)	−0.72 (−2.17,0.73)	0
(b). Matrix of pairwise comparison among intervention methods on TC (shown as mean difference and 95% confidence intervals)					
	PTC	SPD	CUN	LFT	PLB
SUCRA(%)	89.4	64	43	36.6	17
PTC	0	0.47 (−0.60,1.54)	0.68 (-1.79,0.43)	0.75 (−0.32,1.81)	0.88 (−0.03,1.80)
SPD	−0.47 (−0.60,1.54)	0	0.21 (-1.06,0.63)	0.28 (-0.51,1.06)	0.41 (−0.15,0.98)
CUN	−0.68 (-1.79,0.43)	−0.21 (-1.06,0.63)	0	0.06 (-0.77,0.90)	0.20 (-0.43,0.83)
LFT	−0.75 (−0.32,1.81)	−0.28 (-0.51,1.06)	−0.06 (-0.77,0.90)	0	0.14 (-0.41,0.68)
PLB	−0.88 (−0.03,1.80)	−0.41 (−0.15,0.98)	−0.20 (-0.43,0.83)	−0.14 (-0.41,0.68)	0
(c). Matrix of pairwise comparison among intervention methods on LDL (shown as mean difference and 95% confidence intervals)					
	PTC	CUN	LFT	PLB	SPD
SUCRA(%)	84.2	69.5	51.7	30.1	14.5
PTC	0	0.26 (−1.14, 0.62)	0.40 (−0.45,1.25)	0.52 (−0.26,1.30)	0.63 (−0.22,1.48)
CUN	−0.26 (−1.14,0.62)	0	0.14 (−0.39, 0.67)	0.26 (−0.15, 0.66)	0.37 (−0.16,0.90)
LFT	−0.40 (−0.45,1.25)	−0.14 (−0.39,0.67)	0	0.12 (−0.22, 0.46)	0.23 (−0.71,0.25)
PLB	−0.52 (−0.26,1.30)	−0.26 (−0.15,0.66)	−0.12 (−0.22,0.46)	0	0.12 (−0.45, 0.22)
SPD	−0.63 (−0.22,1.48)	−0.37 (−0.16,0.90)	−0.23 (−0.71,0.25)	−0.12 (−0.45,0.22)	0

NAFLD, non-alcoholic fatty liver disease; PTC, probiotics; CUN, curcumin; LFT, Lifestyle; PLB, Placebo; SPD, Special Diet; SUCRA, Surface under the cumulative ranking curve; ALT, Alanine aminotransferase; TC, Total cholesterol; LDL, low-density lipoprotein cholesterol.

### 3.4 Pairwise meta-analysis

**FIGURE 3:**
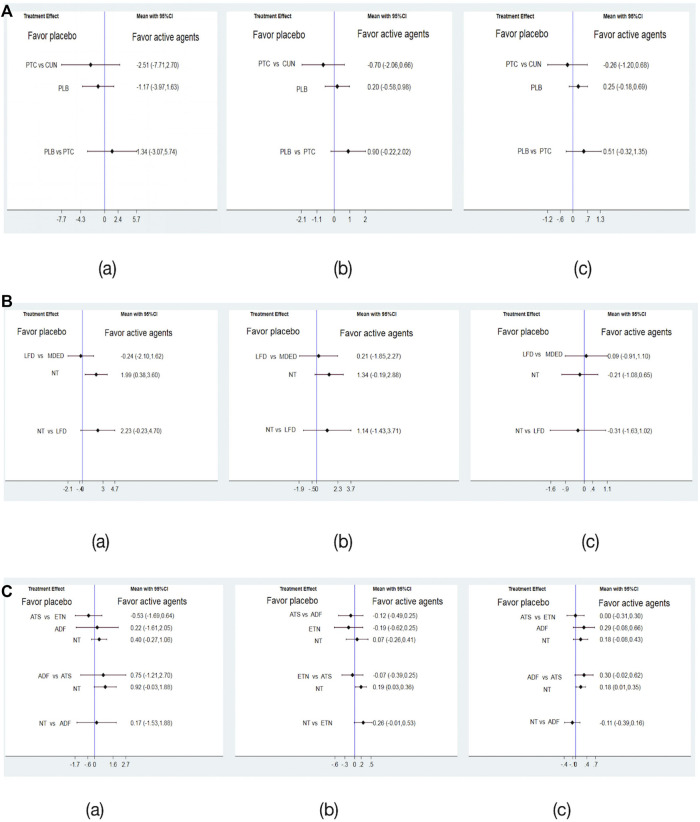
**(A)** Forest plot effcacy of medication with placebo (PLB), (a)Forest plot effcacy of curcumin (CUN) and probiotics (PTC) with placebo in improving alanine aminotransferase (ALT), (b)Forest plot effcacy of curcumin (CUN) and probiotics (PTC) with placebo (PLB) in improving total cholesterol (TC), (c) Forest plot effcacy of curcumin (CUN) and probiotics (PTC) with placebo (PLB) in improving low-density lipoprotein cholesterol (LDL). **(B)** Forest plot effcacy of diet with control, (a) Forest plot effcacy of mediterranean diet (MDED) and low-fat diet (LFD) with not have any type of treatment (NT) in improving alanine aminotransferase (ALT), (b) Forest plot effcacy of mediterranean diet (MDED) and low-fat diet (LFD) with not have any type of treatment (NT) in improving total cholesterol (TC), (c) Forest plot effcacy of mediterranean diet (MDED) and low-fat diet (LFD) with not have any type of treatment (NT) in improving low-density lipoprotein cholesterol (LDL).**(C)** Forest plot effcacy of lifestyle with control, (a) Forest plot effcacy of exercise training (ETN), accept theoretical suggestions (ATS) and alternate-day fasting (ADF) with not have any type of treatment (NT) in improving alanine aminotransferase (ALT), (b) Forest plot effcacy of exercise training (ETN), accept theoretical suggestions (ATS) and alternate-day fasting (ADF) with not have any type of treatment (NT) in improving total cholesterol (TC), (c) Forest plot effcacy of exercise training (ETN), accept theoretical suggestions (ATS) and alternate-day fasting (ADF) with not have any type of treatment (NT) in improving low-density lipoprotein cholesterol (LDL).

**FIGURE 4:**
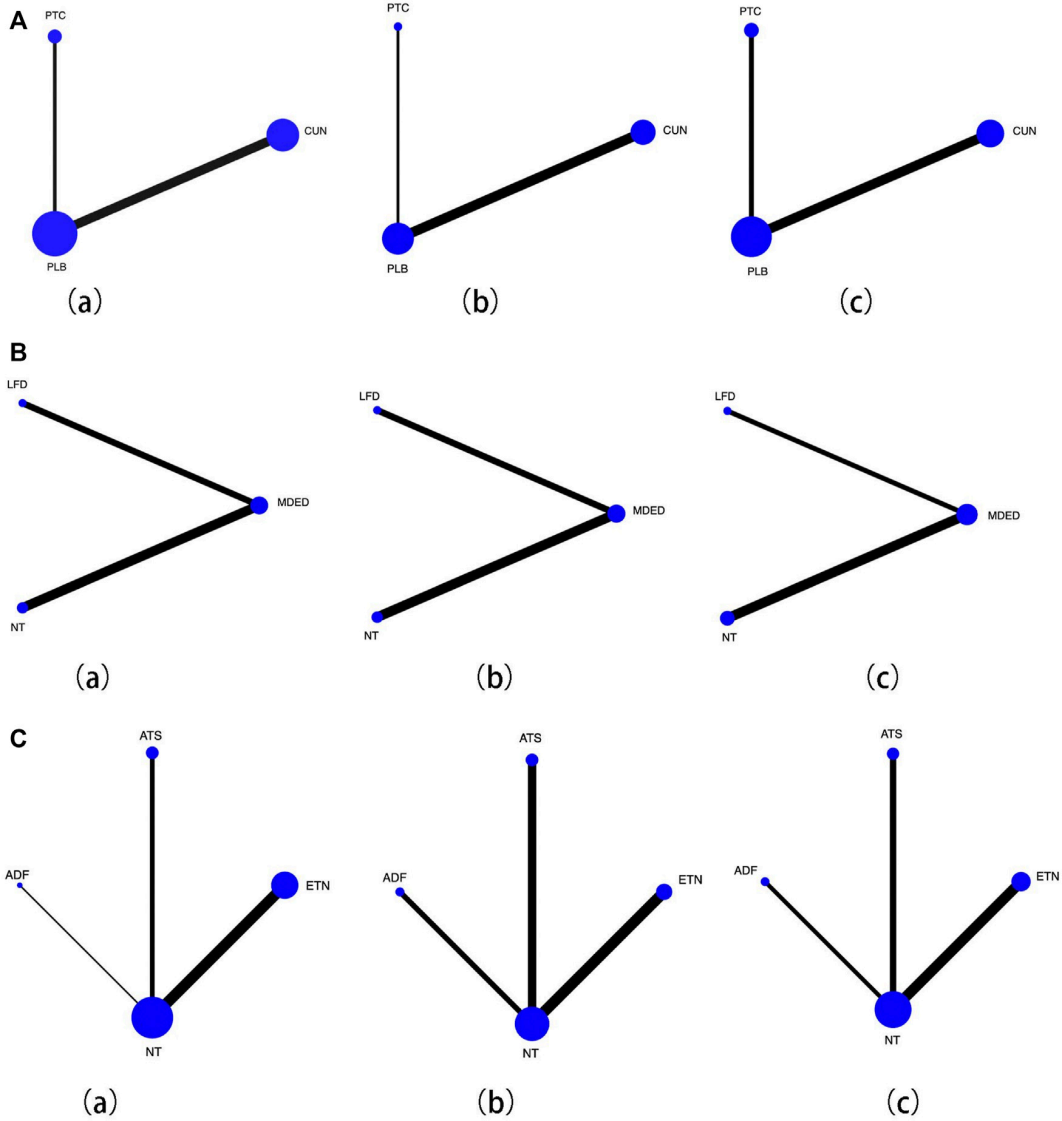
**(A)** Network map for effcacy of medication with placebo (PLB), (a) Forest plot effcacy of curcumin (CUN) and probiotics (PTC) with placebo in improving alanine aminotransferase (ALT), (b) Forest plot effcacy of curcumin (CUN) and probiotics (PTC) with placebo (PLB) in improving total cholesterol (TC), (c) Forest plot effcacy of curcumin (CUN) and probiotics (PTC) with placebo (PLB) in improving low-density lipoprotein cholesterol (LDL). Lines connect the interventions that have been studied in head-to-head comparisons in eligible RCTs. The width of the lines represents the total number of RCTs for each pairwise comparison. The size of each node is proportional to the number of randomized participants. **(B)** Network map for effcacy of diet with control, (a) Forest plot effcacy of mediterranean diet (MDED) and low-fat diet (LFD) with not have any type of treatment (NT) in improving alanine aminotransferase (ALT), (b) Forest plot effcacy of mediterranean diet (MDED) and low-fat diet (LFD) with not have any type of treatment (NT) in improving total cholesterol (TC), (c) Forest plot effcacy of mediterranean diet (MDED) and low-fat diet (LFD) with not have any type of treatment (NT) in improving low-density lipoprotein cholesterol (LDL). Lines connect the interventions that have been studied in head-to-head comparisons in eligible RCTs. The width of the lines represents the total number of RCTs for each pairwise comparison. The size of each node is proportional to the number of randomized participants. **(C)** Network map for effcacy of lifestyle with control, (a) Forest plot effcacy of exercise training (ETN), accept theoretical suggestions (ATS) and alternate-day fasting (ADF) with not have any type of treatment (NT) in improving alanine aminotransferase (ALT), (b) Forest plot effcacy of exercise training (ETN), accept theoretical suggestions (ATS) and alternate-day fasting (ADF) with not have any type of treatment (NT) in improving total cholesterol (TC), (c) Forest plot effcacy of exercise training (ETN), accept theoretical suggestions (ATS) and alternate-day fasting (ADF) with not have any type of treatment (NT) in improving low-density lipoprotein cholesterol (LDL). Lines connect the interventions that have been studied in head-to-head comparisons in eligible RCTs. The width of the lines represents the total number of RCTs for each pairwise comparison. The size of each node is proportional to the number of randomized participants.

We subdivided medication, diet and lifestyle intervention measures and then conducted a pairwise comparison of ALT, TC and LDL. The medication subdivisions were CUN, PTC and PLB, and there were no significant differences in improvements in ALT, TC and LDL levels Figure 3A(a–c), and the network map were shown in Figures 4A(a–c). The diet subdivisions were MDED, low-fat diet (LFD), and no treatment (NT). As shown in Figure 3Ba, MDED was significantly better than NT in improving ALT [SMD 1.99, 95% CI (0.38, 3.60)]. There was no significant difference in improving TC and LDL Figure 3B(b, c), and the network map were shown in Figure 4B(a–c). The lifestyle subdivisions were exercise training (ETN), acceptance of theoretical suggestions (ATS), alternate-day fasting (ADF) and NT. There was no significant difference in improvements to ALT, as shown in Figure 3C(a). In terms of improving TC and LDL, ATS was significantly better than NT [SMD 0.19, 95% CI (0.03, 0.36)] [SMD 0.18, 95% CI (0.01, 0.35)] Figure 3C(b, c), and the network map were shown in Figure 4C(a–c).

### 3.5 Sensitivity and publication bias

The sensitivity analysis showed that any single study or cluster study with certain characteristics had little effect on the change in SMD and its corresponding 95% CI. Significant publication bias was not reported by Egger’s regression test or Begg’s adjusted rank correlation test.

## 4 Discussion

To the best of our knowledge, this study was the first to evaluate various interventions for NAFLD using pairwise comparisons and network meta-analyses to explore the effects of different types of interventions on liver cell damage in NAFLD, the impact on lipid metabolism and the relative efficacy of reducing the content of blood lipids and cholesterol. The most important finding of this study was that compared with diet and lifestyle, the use of probiotics had the best effect on improving NAFLD indicators, while MDED and ATS were superior to no treatment.

CUN, a natural polyphenol compound extracted from turmeric, exhibits oxidative activity by removing ROS, helps to prevent and resolve liver damage, and has a protective effect on the liver (Yarru et al., 2009). CUN inhibits adipocyte differentiation by inhibiting PPAR-γ and increasing adenosine monophosphate-activated protein kinase, thereby reducing body fat mass and leading to increased lipolysis (Bradford, 2013). In our NMA, there was no significant difference in the impact of CUN on ALT, TC and LDL compared with that of PLB and PTC. However, SUCRA ranked CUN as performing better than the placebo group in terms of improving TC and LDL. Several previous studies have reported conflicting effects of CUN on body composition in NAFLD patients (Song et al., 2022). One study reported (Chen et al., 2021) that CUN had no significant effect on body mass index (BMI), body weight, or waist circumference (WC). Another study (Mousavi et al., 2020) showed that the use of different doses of CUN and the long duration of the trial could affect BMI and WC in patients with NAFLD. Therefore, we found that due to the difference in sample sizes of the included studies, different dosages and durations of medication resulted in heterogeneity.

PTCs are acquired symbiotic microorganisms that are beneficial to host health when consumed in sufficient quantities (Pan et al., 2022). PTC can treat NAFLD by regulating the composition of the intestinal flora and the production of antibacterial factors, changing the permeability and function of intestinal epithelial cells, modifying endotoxemia, inhibiting inflammation, and regulating the immune system. (Hecht et al., 2016). At the time of our analysis of the included studies, PTC was not significantly different from CUN and PLB, but SUCRA indicated that PTC was the best intervention for improving ALT, TC, and LDL. Adverse effects of probiotics are rare, except for changes in flatulence or bowel habits, but one study (Borriello et al., 2003) suggested that additional use should be carefully evaluated in immunocompromised or critically ill patients to limit the risk of endocarditis or sepsis. In addition, *Lactobacillus* is considered an emerging pathogen due to its clinically important resistance to the antibiotic vancomycin (Sponton et al., 2020). The PTCs used to treat NAFLD mainly include *Lactobacillus* acidophilus, *Lactobacillus* rhamnosus, *Streptococcus* paralactis, *Lactobacillus* pentosus, *Lactobacillus* lactis and *Lactobacillus* brevis. A new type of called “NGPs” has also been applied, including the mucobacter Akkermansia A. muciniphila, faecalis F. praussnitzii and *Bacteroides* B. fragilis (Sáez-Lara et al., 2016).

The mediterranean diet (MDED), a healthy eating pattern with a diet low in saturated fats and animal protein, high in antioxidants, adequate fiber, monounsaturated fatty acids, and a balance of omega-3 and omega-6 fats, generally consists of a high consumption of whole grains, fruits, legumes, vegetables, nuts, moderate amounts of dairy products, and a moderate consumption of red wine (Cheng et al., 2020). Studies have shown that MDED can reduce the development risk of NAFLD through the nutritional effects of its bioactive compounds and the antioxidant and anti-inflammatory effects of the phytochemicals it contains (Sato and Sassone-Corsi, 2022). MDED not only positively improves inflammatory biomarkers but also improves clinical parameters such as body weight, waist circumference, liver fat accumulation, blood drug level transaminase, glutamyltransferase, triglycerides, cholesterol, insulin, and insulin resistance (Hunter, 2019). Our NMA shows that MDED can significantly improve the indicators of NAFLD compared with no intervention. MDED is appropriate for individuals who are not fit for exercise and patients who have gastrointestinal intolerance to drugs.

As first-line treatment measures, health education and physical activity can reduce the level of liver enzymes, improve fatty liver, and reduce the content of triglycerides in the liver and markers of liver cell injury in patients with NAFLD, with the benefits of low cost and high return (Hackstein and Klenerman, 2020). High-intensity interval training and aerobic exercise can significantly reduce ALT, AST, triglycerides and other indicators in patients with NAFLD (Wang et al., 2019). One study showed that patients who receive dietary and exercise advice from a nutritionist and exercise physiologist, respectively, and were encouraged to follow the advice, including controlling total calorie intake, changing sedentary lifestyles, and choosing appropriate exercise for their preferences, could effectively improve their symptoms and indicators of NAFLD (Umpierre et al., 2011). In our study, patients who received health education had significantly different results than those who received no intervention, suggesting an important role for advice and intervention in the lifestyle of NAFLD patients. However, the difference in exercise mode and intensity may be a source of the greater heterogeneity.

## 5 Limitations

First, there was substantial heterogeneity in this NMA due to the large number of studies included in our study, patients with different dosages and durations of medication, exercise methods, exercise times and exercise. Although we conducted subgroup analyses, the studies did not mention individual age, gender, exercise intensity, exercise time, exercise facilities and other contents, which severely limited the availability of data. Second, the drugs we included were only natural extracts and probiotics, which cannot represent the efficacy and safety of all drugs in terms of treatment. Finally, only English articles were selected, which may limit the comprehensiveness of the data.

## 6 Conclusion

The network meta-analysis integrated the available evidence from previous studies, summarized several measures to improve NAFLD in terms of drugs, lifestyle and diet, and provided some suggestions for clinicians to choose some treatment methods. Our study shows that probiotics are most likely to be the most effective treatment for improving NAFLD indicators. Professional advice on diet or exercise is more effective in treating NAFLD than no intervention. However, considering the possible limitations of our meta-analysis, the results should be interpreted with caution. More multigroup randomized controlled trials should be conducted in the future to provide more direct evidence of the relative efficacy of different interventions.
